# Functional properties and antioxidant activity of gelatine and hydrolysate from deer antler base

**DOI:** 10.1002/fsn3.1621

**Published:** 2020-05-10

**Authors:** Chang Liu, Yunshi Xia, Mei Hua, Zhiman Li, Lei Zhang, Shanshan Li, Ruize Gong, Songxin Liu, Zeshuai Wang, Yinshi Sun

**Affiliations:** ^1^ Institute of Special Animal and Plant Sciences CAAS Changchun, Jilin People’s Republic of China; ^2^ Jilin Agricultural University Changchun, Jilin People’s Republic of China

**Keywords:** antioxidant, deer antler base, functional properties, hydrolysate

## Abstract

Gelatine was extracted from deer antler base by the hot water method and hydrolyzed with trypsin. A comparison of the properties of gelatine before and after enzymatic hydrolysis showed a decline in the surface hydrophobicity, enhanced thermal stability, broadening of the particle size distribution, a zeta potential shift to a lower pH, reduced foaming and emulsifying properties, and enhanced antioxidant activity. Hydrolysis increased the gelatine antioxidant activity in DPPH and FRAP assays. These results indicate that the functional properties of deer antler base gelatine may be affected by trypsin modification.

## Graphical abstract

The properties of deer antler base gelatin were compared before and after hydrolysis. The surface hydrophobicity declined after hydrolysis. The emulsifying stability of the hydrolysate increased. The antioxidant activity of DPPH and FRAP was enhanced after hydrolysis.

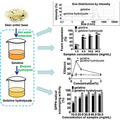

## 1 INTRODUCTION

Deer (Cervidae) are animals mainly distributed in Eurasia, North America, South America and southwest Africa. As in caribou, antlers are found in deer males but not females and periodically fall off and regenerate every year. This special animal organ regeneration model has attracted attention worldwide (Wang et al., 2019; Dong, Coates, Liu, Sun, & Li, 2019; Yao et al., 2019).

Deer antler base is the residual antler that falls off naturally in the spring following ossification. This material has been applied in health care to improve immunity, strengthen bones, relieve stress, for antioxidant activities, etc. (Wu et al., 2013). Deer antler base is typically boiled to obtain a glue solution. As deer antler base glue is mainly composed of collagen (Wei et al., 2015), the glue solution is considered to be a gelatine.

The gelatine and its hydrolysates have attracted increasing attention from researchers because of potential health‐promoting effects (Tkaczewska, Jamróz, Kulawik, Morawska, & Szczurowska, 2019). Deer antler base is mainly composed of proteins, peptides, mineral elements, amino acids, cholesterol, polysaccharides, and lipids (Wang et al., 2015). Proteins, peptides, and amino acids are considered to be the critical health‐promoting ingredients (Guan, Zhao, Wang, Wang, & Li, 2019). Enzymatic hydrolysis is a key means of realizing biological activities and has been the subject of active research. In recent years, studies on deer antler base gelatine have focused on the chemical composition, peptide preparation, and pharmacological effects (Guan et al., 2019; Guan, Zhao, & Li, 2018b). However, for industrial production of deer antler gelatine as a food, the functional properties of deer antler gelatine or gelatine hydrolysate must be studied from a processing perspective in addition to investigating the bioactivity, the extraction efficiency, and stability of the bioactive peptides. However, no information is available on the functional properties of deer antler base gelatine because of the scarcity of relevant studies.

Deer antler base gelatine is rich in nutrition and is extremely beneficial to human health. The aim of this study was to evaluate the following functional properties of deer antler gelatine before and after enzyme hydrolysis: thermal properties, the zeta potential, the particle size distribution, the surface hydrophobicity, foaming properties, and emulsifying properties. The antioxidant activity of gelatine was also analyzed.

## 2 MATERIALS AND METHODS

### 2.1 Materials

Anilinonaphthalene‐8‐sulfonic acid (ANS) was purchased from Sigma‐Aldrich Chemical Co. An amino acid mixture standard of type H was obtained from Woke. Trypsin (250 U/mg, CAS:900‐07‐7) and pepsin (2,500 U/mg, CAS: 9001‐75‐6) were obtained from Sangon. All chemicals were analytical grade and used without further purification. Deer antler base was obtained from Zuojia.

### 2.2 Extraction and hydrolysis of gelatine from deer antler base

Deer antler base gelatine was obtained by a boiling water extraction process, which was a modification of that described by Guan, Zhao, and Li (2018a). The gelatine was boiled in a 20x volume of water three times (for 2 hr each time). After centrifugation, the supernatant was combined and concentrated. The concentrate was freeze‐dried at 50°C in a lyophilizer.

Gelatine hydrolysate was obtained by enzymolysis of the gelatine using trypsin. A prescribed mass of gelatine powder was dissolved in 1,000 g of deionized water at 37°C under constant stirring at 600 rad/min. The pH was adjusted using 1 mol/L NaOH and 1 mol/L HCl. Trypsin and pepsin were added to the sample solutions at pHs of 8.0 and 2.0, respectively. The hydrolysis reaction was terminated by heating at 85°C for 15 min. The hydrolysate reaction liquid was freeze‐dried.

### 2.3 Degree of hydrolysis

The degree of hydrolysis (DH) was determined using the ninhydrin method of Huang, Ruan, Qin, Li, and Zheng (2017).

### 2.4 Molecular weight distribution

The Mw distribution was determined by the method of Chun, Jo, Min, and Hong (2014) with some modifications. The pretreated deer antler gelatine and its hydrolysate were centrifuged at 10,000 rad for 5 min. The supernatant was performed on a gel permeation chromatography (GPC) (ELEOS System, Wyatt) equipped with two Shodex OHpak SB‐806 and SB‐803 columns. The mobile phase was distilled/deionized water and 0.02 wt% NaN_3_ at a flow rate of 1 ml/min, and the Mw distributions were monitored using a laser detector and differential detector. Molecular weight distribution was calculated as Mw/Mn.

### 2.5 Amino acid analysis

The amino acid composition of the samples was determined using the procedure described by Wang et al. (2017) with an amino acid analyzer (L‐8900A, Hitachi). The samples were hydrolyzed at 110°C for 22 hr in 6 mol/L HCl.

### 2.6 Surface hydrophobicity

The surface hydrophobicity of the deer antler base gelatine and gelatine hydrolysate was determined using the ANS fluorescent probe method described by Mad‐Ali, Benjakul, Prodpran, and Maqsood (2016). The fluorescence intensity was tested using a Hitachi F‐2500 fluorescence spectrometer (Hitachi, Ltd.) at an excitation wavelength of 390 nm, an emission wavelength of 510 nm, and a scan speed of 300 nm/min.

### 2.7 Zeta potential and particle size distribution

The zeta potential and the particle size distribution were measured according to Tkaczewska et al. (2019). The particle size distribution of the deer antler base gelatine and the gelatine hydrolysate was measured via dynamic light scattering using a Zetasizer Nano ZS Malvern instrument. The zeta potential was determined using a Zetasizer Nano ZS Malvern apparatus.

### 2.8 Thermal properties

A thermal analysis of the deer antler base gelatine and hydrolysate was carried out by differential scanning calorimetry (DSC) (TA, Q20) according to Al‐Ruwaih, Ahmed, Mulla, and Arfat (2019). Samples weighing approximately 8 mg were subjected to heating with a ramp up from 0 to 150°C at 10°C/min under a nitrogen atmosphere (flow rate, 50 ml/min) to prevent oxidative reactions. Temperature and heat capacity calibration of the DSC was performed using indium and sapphire, respectively. An empty aluminum pan was used as a reference.

### 2.9 Foaming capacity and foaming stability

The foaming capacity (FC) and foaming stability (FS) of deer antler base gelatine and gelatine hydrolysate were measured using a slight modification of the method of Ahmed, Al‐Ruwaih, Mulla, and Rahman (2018). Ten milliliters of the sample solution (1.0–30.0 mg/ml) was blended using a homogenizer (ULTRA‐TURRAX, T25 Digital IKA‐Werke Stuttgart Staufen) at 15,000 rad/min for 1 min. The total volume after whipping was determined several times over 0–60 min. The FC was determined as the foam expansion at 0 min, and the FS was determined as the foam expansion from 0 to 60 min. The foam expansion formula is given below:

(1) $$\text{Foam expansion}\left(\%\right)=\left(\frac{V-V_{0}}{V}\right)\times 100$$

where *V_0_* and *V* are the volumes before and after homogenization (ml), respectively.

### 2.10 Emulsifying properties

The emulsifying activity index (EAI) and the emulsion stability index (ESI) were measured by modifying the method of Rajabzadeh, Pourashouri, Shabanpour, and Alishahi (2018). Dispersions containing 5 ml of soybean oil and 15 ml of gelatine or hydrolysate solution (1.0–30.0 mg/ml) were homogenized at 20,000 rad/min for 1 min. Then, 50 μL of the emulsion was pipetted from the bottom of the mixture at 0 and 10 min and diluted with a sodium phosphate buffer (pH 7.0) containing 1.0 mg/ml SDS. The absorbance of the diluted emulsion was measured at 500 nm after vortexing. The EAI and ESI were calculated using the following equations:

(2) $$\text{EAI}\left(m^{2}/g\right)=\frac{2\times 2.303\times A_{0}\times N}{c\times \varphi \times \theta \times 10^{4}}$$

(3) $$\text{ESI}\left(\min\right)=\left(\frac{A_{0}}{\Delta A}\right)\times t$$

where *A*_0_ and *A* are the absorbance values measured at 0 and 10 min of emulsion formation (500 nm), respectively; *N* is the dilution ratio; *c* is the concentration of gelatine or hydrolysate (g/mL); φ is the path length of the cuvette; and *θ* is the oil ratio.

### 2.11 Determination of antioxidant activities

The DPPH, ABTS, and FRAP radical scavenging activities were determined using a modification of the methods of Xie et al. (2017). A total of 19.6 mg of DPPH were dissolved in 100 ml of ethanol to form a DPPH stock solution, which was stored in the dark. Twenty milliliters of 7 mmol/L ABTS solution and 352 μL of 140 mmol/L potassium persulfate were mixed to form an ABTS stock solution, which was stored in the dark overnight. Before use, the two stock solutions were diluted with ethanol to produce working solutions. The absorbances of the DPPH and ABTS working solutions were adjusted to 0.70 ± 0.05 (525 and 734 nm, respectively). Then, 100 ml of a 300 mmol/L sodium acetate buffer (pH 3.6), 10 ml of 10 mmol/L tripyridyltriazine, and 10 ml of 20 mmol/L ferric chloride were mixed to form the FRAP working solution, which was stored in the dark. Next, 2.0 ml of the sample solution was mixed with 2.0 ml of the DPPH working solution and stored in the dark. After 30 min, the absorbance was tested at 525, 734, and 593 nm. The standard curve for the FRAP assay was drawn using ferrous sulfate solution. Each sample was tested in triplicate.

### 2.12 Statistical analysis

The data were expressed as the mean ± standard error and evaluated for statistical significance using SPSS software (Version 19.0). The mean values were compared using the one‐way analysis of variance (ANOVA). Differences at *p* < .05 were considered significant.

## 3 RESULTS AND DISCUSSION

### 3.1 DH and molecular weight distribution of the deer antler base gelatine hydrolysate

**FIGURE 1:**
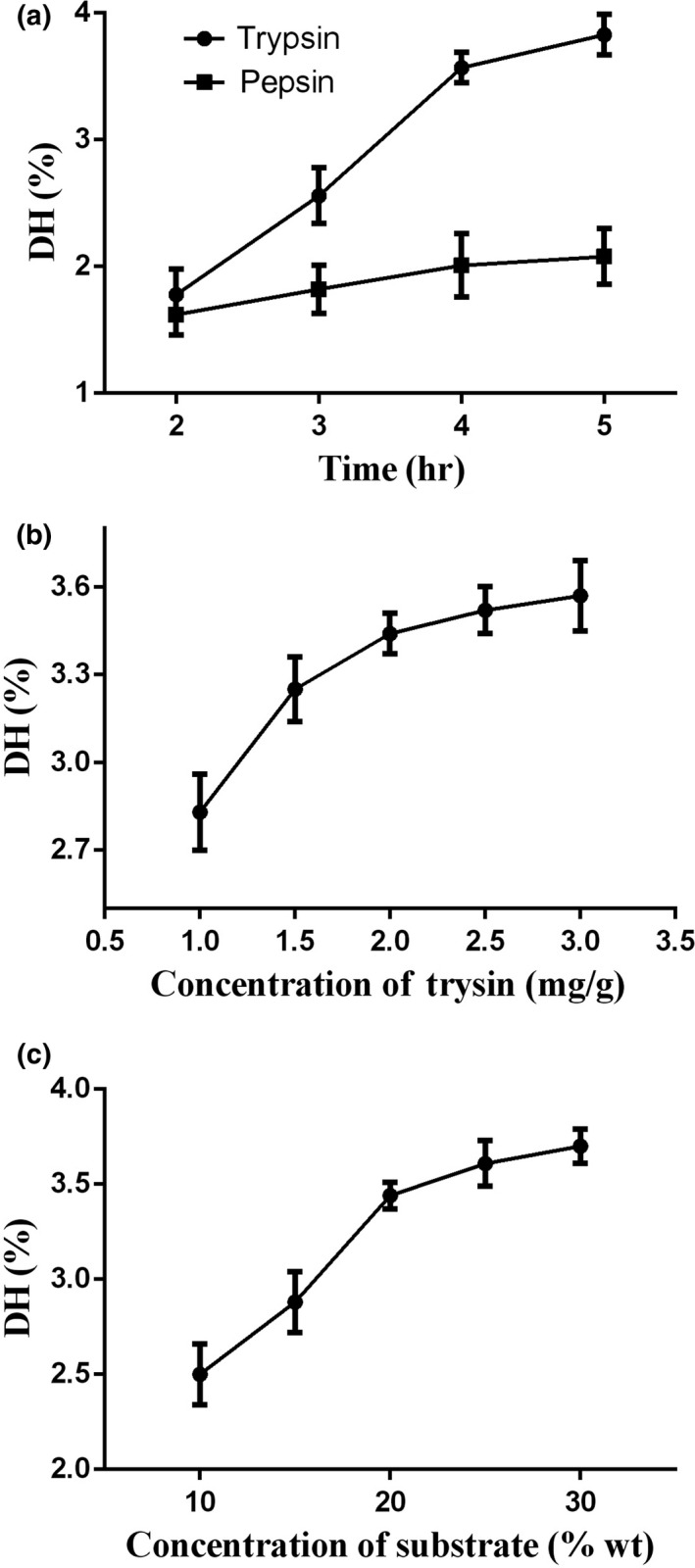
Selection of enzymolysis conditions based on degree of hydrolysis (DH): in (a), the trypsin and pepsin contents are 3.0 mg/g sample and 0.3 mg/g sample, respectively; experimental conditions: 2.0 mg/g trypsin, a reaction time of 4 hr, 20 wt% substrate, 37°C, and pH 8.0 (except for the factor under investigation)

The enzyme species and concentration and the substrate were investigated by monitoring the hydrolysate DH. As shown in Figure 1a, both trypsin and pepsin increased the hydrolysate DH, which grew slowly after 4 hr. Trypsin had a stronger effect on the enzymatic reaction of gelatine than pepsin and was therefore selected for subsequent experiments. As shown in Figure 1b, the 2.0‐mg/g trypsin sample was the most effective of the various trypsin concentrations that were investigated, and addition of higher quantities of trypsin produced lower increases of DH. Figure 1c shows that the DH increased with the substrate concentrations up to 20 wt% substrate and did not increase significantly for higher substrate concentrations. Thus, a 20‐wt% substrate concentration was selected for subsequent experiments.

Trypsin is an endopeptidase that can cleave the carboxyl (COOH) of lysine and arginine residues in the polypeptide chain. A reaction pH of 8.0 and a 4‐hr treatment time were selected. The DH is shown in Table 1. As the water boiling treatment partially hydrolyzed the protein macromolecules during gelatine extraction, a low DH of 1.61% was obtained for the gelatine. The DH increased to 3.44% after enzyme hydrolysis.

**TABLE 1:** Degree of hydrolysis, surface hydrophobicity, thermal stability, and zeta potential of deer antler base gelatine and its hydrolysate

	DH (%)	*S*_0_	*T*_d_ (°C)	Zeta potential	Mw (10^4^ g/mol)	Mw/Mn
Gelatin	1.61 ± 0.00^b^	4.05 ± 0.02^a^	110.3 ± 1.32^b^	3.22 ± 0.01^a^	3.60 ± 0.00^a^	1.66 ± 0.00^a^
Gelatin hydrolysate	3.44 ± 0.01^a^	3.24 ± 0.01^b^	117.9 ± 1.06^a^	2.30 ± 0.01^b^	2.70 ± 0.01^b^	2.78 ± 0.01^b^

Values are means ± standard deviation. Different letters in the same row indicate significant differences (*p* < .05).

Abbreviation: DH, degree of hydrolysis.

Gel permeation chromatography provides information regarding the Mw and molecular weight distribution of deer antler base gelatine as shown in Table 1. After enzyme hydrolysis of trypsin, molecular weight lowered from 3.60 to 2.70 × 10^4^ g/mol, and the molecular weight distribution become wider from 1.66 to 2.78. That is because enzymatic hydrolysis causes the breaking of the molecular chain, and the result was consistent with Liu et al. (2020).

### 3.2 Amino acid analysis

Table 2 shows the amino acid composition of deer antler base gelatine and its hydrolysate. A total of 17 amino acids were identified and quantified, including the following essential amino acids for the human body: lysine, phenylalanine, methionine, threonine, isoleucine, leucine, valine, and histidine. Tryptophan was destroyed by the pretreatment of the amino acids by acid hydrolysis and was therefore not detected. All the amino acids were rich in glycine acids before and after hydrolysis and are classified as umami amino acids. This result was consistent with those for gelatine from bones, such as chicken keel bone (Cordeiro et al., 2020) and skipjack tuna bone (Yang, Zhao, Qiu, Chi, & Wang, 2019). Some amino acids, such as aspartic acid, serine, and glutamic acid, were sensitive to hydrolysis and showed a considerable decrease in concentration. Other amino acids, such as cysteine, lysine, histidine, and all the hydrophobic amino acids, controlled the emulsification properties of the emulsified products (Cordeiro et al., 2020).

**TABLE 2:** Amino acid composition of deer antler base gelatine and hydrolysate (residues/1000 residues)

Amino acids	Gelatine	Gelatine hydrolysate
Aspartic acid	76.68 ± 0.08^a^	39.56 ± 0.07^b^
Threonine	28.15 ± 0.10^a^	25.25 ± 0.02^b^
Serine	40.65 ± 0.08^a^	20.44 ± 0.02^b^
Glutamic acid	131.83 ± 0.04^a^	46.93 ± 0.04^b^
Glycine	274.93 ± 0.33^a^	308.23 ± 0.50^b^
*Alanine	123.98 ± 0.08^a^	159.12 ± 0.28^b^
Cysteine	2.77 ± 0.02^a^	7.37 ± 0.11^b^
*Valine	33.58 ± 0.03^a^	42.24 ± 0.06^b^
*Methionine	2.17 ± 0.02^a^	7.12 ± 0.07^b^
*Isoleucine	18.46 ± 0.05^a^	21.57 ± 0.12^b^
*Leucine	46.37 ± 0.09^a^	54.07 ± 0.22^b^
*Tyrosine	10.89 ± 0.04^a^	13.40 ± 0.03^b^
*Phenylalanine	30.51 ± 0.03^a^	35.26 ± 0.22^b^
Lysine	51.84 ± 0.02^a^	72.81 ± 0.26^b^
Histidine	7.29 ± 0.02^a^	11.87 ± 0.06^b^
Arginine	10.06 ± 0.26^a^	9.94 ± 0.33^a^
*Proline	109.84 ± 0.06^a^	124.81 ± 0.22^b^
Total	1,000	1,000

*Hydrophobic amino acids.

Results are expressed as mean ± standard deviation from triplicate determinations; different letters in same row indicate significant differences (*p* < .05).

### 3.3 Surface hydrophobicity

The surface hydrophobicity (*S*_0_) is a measure of the number of hydrophobic groups on the surface of proteins. This index is also an important means of following changes in the protein molecular structure (Pinholt et al., 2010). Zhang and Lu (2015) found that a large quantity of hydrophobic groups was exposed under the enzyme hydrolysis of proteins and subsequently aggregated and cross‐linked, leading to a decline in protein hydrophobicity. The *S*_0 _of the gelatine and its hydrolysate are shown in Table 1. Gelatine had a *S*_0_ of 4.05 before enzyme hydrolysis, which was reduced to 3.24 after enzyme processing. This result was consistent with the abovementioned results from the literature. However, the *S*_0 _values of the gelatine and its hydrolysate were much lower than those of other proteins, such as bacteriorhodopsin, bovine serum albumin, and ovalbumin (Cardamone & Puri, 1992), which may explain the naturally high hydrophilicity of the gelatine. Mad‐Ali et al. (2016) reported *S*_0 _values of 3.49, 2.35, and 1.78 for spray‐dried goat skin gelatine, its freeze‐dried counterparts, and commercial bovine gelatine, respectively, which are similar to the results obtained in this study.

### 3.4 Zeta potential and size distribution

**FIGURE 2:**
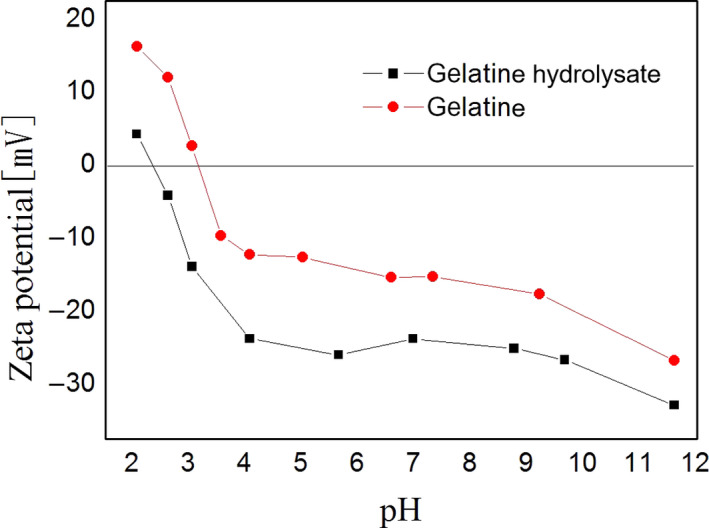
Zeta potential of deer antler base gelatine and its hydrolysate in aqueous solution as a function of pH

The isoelectric point (IEP) can be determined from the zeta potential. The IEP is the pH value of a particle, colloid, or molecule with a zero net charge at the shear plane. At this pH, a colloidal particle with a zero zeta potential remains stationary in the electric field. Particles near this pH lose electrostatic stability and have a tendency to conglomerate (Ai, Guo, Zhou, Wu, & Jiang, 2018). The zeta potential of the gelatine and its hydrolysate are shown in Figure 2, and the zeta potential values are shown in Table 1. The zeta potential of the gelatine solution increased from −26.8 to 16.2 mV with a reduction in pH from 11.6 to 2.1 because of protonation of amino and COOH groups. At lower pH, the gelatine is positively charged because of protonation of amino moieties (NH_2_) to produce ammonium groups $\left({\text{NH}}_{4}^{+}\right)$ . Between pH values of 2.5 and 3.0, some elements of the COOH group become negatively charged, decreasing the surface charge. At a pH of 3.21, the zeta potential is zero, corresponding to the IEP. The amino moieties and COOH groups dissociate into anions and cations to equal extents and in the same manner, such that the macromolecules are neutral. At pHs above the IEP, the COOH ionize into carboxylate groups (COO^−^), resulting in negatively charged gelatine macromolecules (Yang, Anvari, Pan, & Chung, 2012). During gelatine hydrolysis, molecular rearrangement changes the native distribution of the protein surface charge. Higher electrostatic repulsion forces among hydrolysate particles may improve solubility and other related properties (Zhang et al., 2017). The zeta potential values of the gelatine hydrolysate varied between 3.22 mV at pH 2.1 and −32.9 mV at pH 11.6, and the IEP was at pH 2.30. The zeta potential of the gelatine hydrolysate was more negative than before hydrolysis, which may be attributed to the liberation of COOH groups, because enzymatic hydrolysis resulted in dissociation above the IEP (Tamm, Herbst, Brodkorb, & Drusch, 2016). A similar phenomenon was observed for pea protein hydrolysates (Wouters et al., 2017) and wheat gluten hydrolysates (Tamm et al., 2016).

**FIGURE 3:**
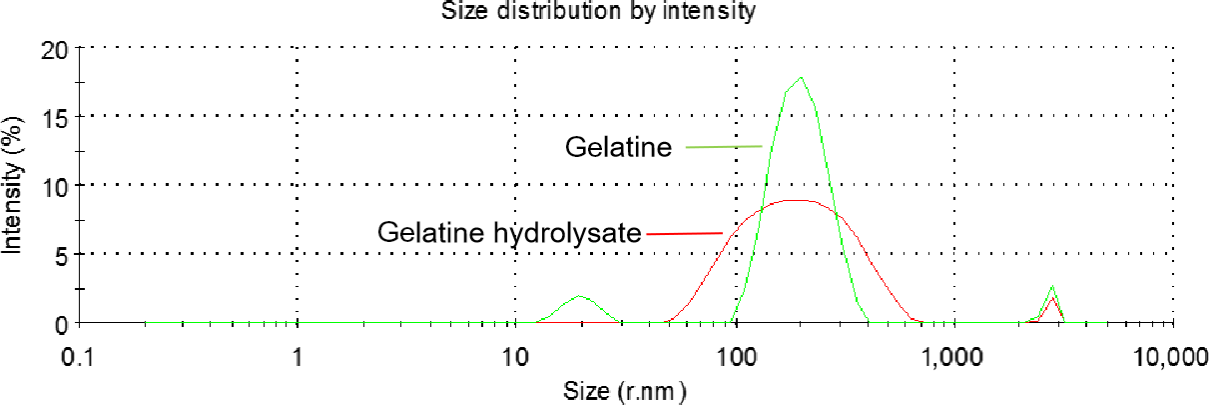
Particle size distribution of deer antler base gelatine and its hydrolysate

The particle size distribution of the gelatine and its hydrolysate are shown in Figure 3. The gelatine particle sizes were in the 100–400 nm range. After treating the gelatine with the enzyme, the particle size distribution broadened to 50–700 nm, indicating the formation of larger aggregates and smaller particles. Aggregation during enzyme modification could result from hydrophobic interactions that induce changes in the surface hydrophobicity. These results were in agreement with those of Tkaczewska et al. (2019) for gelatine and hydrolysate from carp skin, wherein the particle size distribution broadened after hydrolysis.

### 3.5 Thermal properties

**FIGURE 4:**
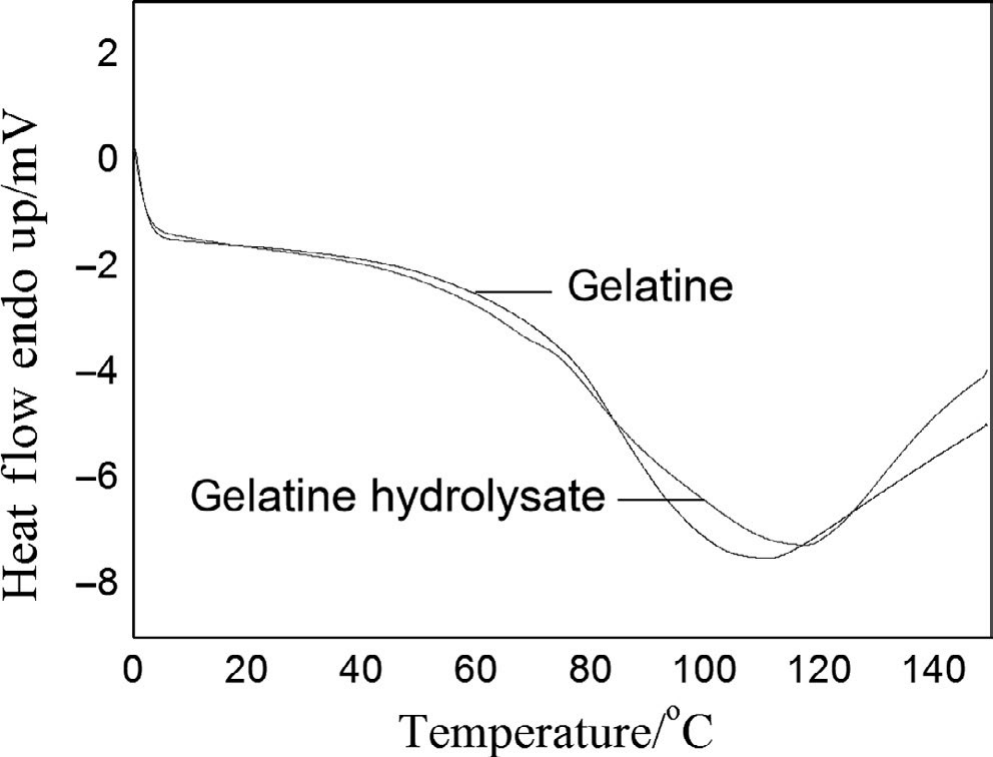
DSC curves of deer antler base gelatine and its hydrolysate. DSC, differential scanning calorimetry

The thermal properties of the gelatine and its hydrolysate were determined by DSC. In Figure 4, each sample has a prominent endothermic peak, which corresponds to the thermal denaturation of protein. The thermal denaturation temperature (*T*_d_) of the gelatine was 110.3°C and increased to 117.9°C after hydrolysis, which could be attributed to the formation of more stable regions by structural destruction and reorganization during enzyme modification. The specific mechanism remains to be determined. Enzyme hydrolysis improves thermal stability.

### 3.6 Foaming properties

**FIGURE 5:**
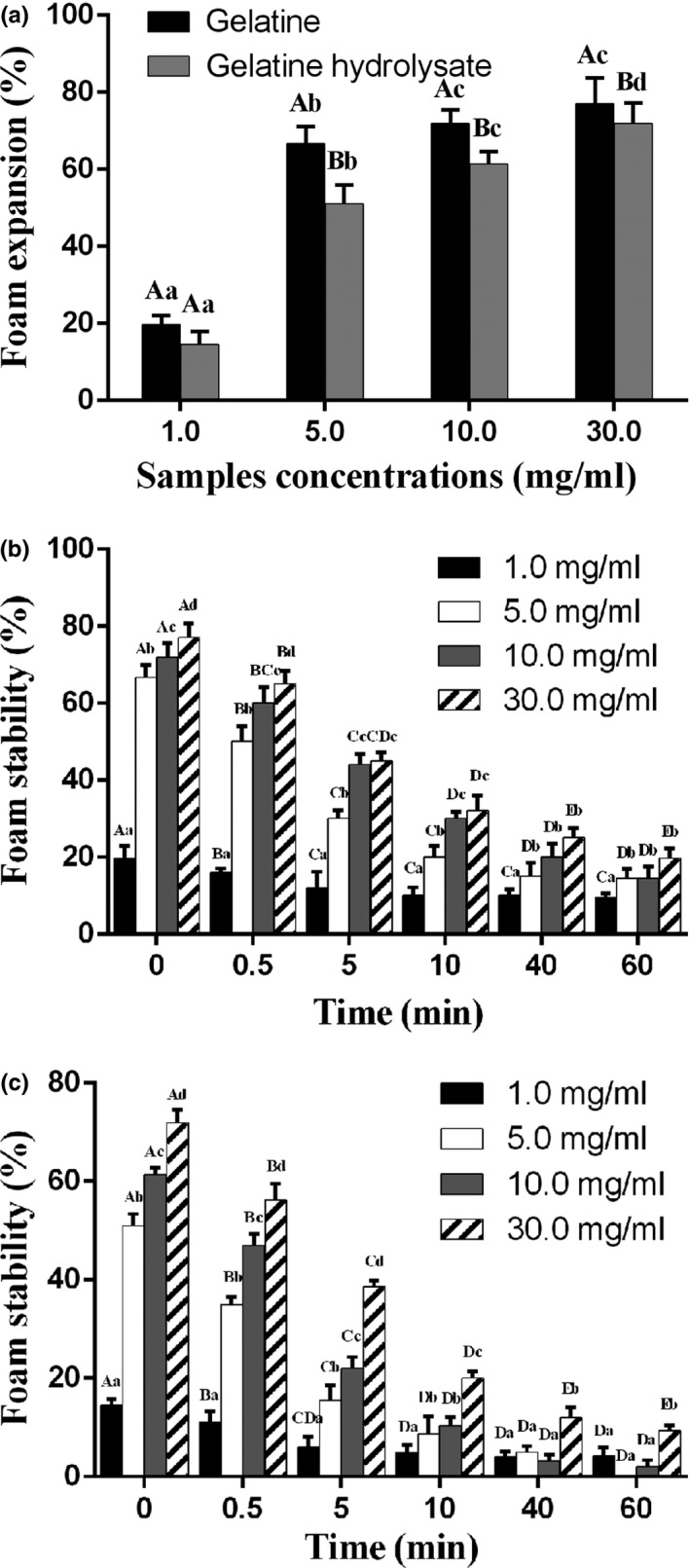
Foaming properties of deer antler base gelatine and its hydrolysate at various concentrations (1.0–30.0 mg/ml): (a) foam expansion; (b) foaming stability of gelatine; (c) foaming stability of hydrolysate; bars represent standard errors (*n* = 3); columns with lowercase (uppercase) letters indicate significant difference (*p* < .05) for the same group (concentration) for different concentrations (groups)

Figure 5 shows how different concentrations of the gelatine and its hydrolysate affected the foaming properties of aqueous compounds. The higher concentrations investigated appear to significantly enhance foam expansion (*p* < .05). The aqueous gelatine showed a high FC at all the tested concentrations (1.0–30.0 mg/ml), as shown in Figure 5a (*p* < .05). The FC has been correlated with peptide size; that is, small peptides can easily migrate into the air–water interface (Rajabzadeh et al., 2018). However, our results showed that gelatine with a low DH (1.61%) had a high FC, which could be attributed to enhanced support of the foam by the higher‐viscosity aqueous gelatine than the aqueous hydrolysate at the same concentration. The FC increased with the concentrations of the gelatine and its hydrolysate. The FC values of the gelatine and its hydrolysate were lower than that of Nile tilapia bone (Tinrat & Sila‐Asna, 2017) and bovine bone (Arioui, 2018).

Higher concentrations of the gelatine and its hydrolysate improved FS, as shown in Figure 5b,c. The FS decreased markedly with time. Over 60 min, the FS of the gelatine (19.8%‐77.1%) was higher than that of its hydrolysate (9.4%‐71.9%) at the same concentration of 30.0 mg/ml. Proteins with good foaming abilities can migrate rapidly into the air–water interface, unfold and rearrange at the interface (Halling, 1981). Foam formation is controlled by three factors: transport, penetration, and structural modification of protein molecules at the air–water interface (Klompong, Benjakul, Kantachote, & Shahidi, 2007). Peptides with long chains formed thicker and stronger films surrounding air bubbles than those with short chains. These results were consistent with that of Intarasirisawat, Benjakul, Visessanguan, and Wu (2012) that hydrolysate with a low DH and a long chain yielded a highly stable foam.

### 3.7 Emulsifying properties

**FIGURE 6:**
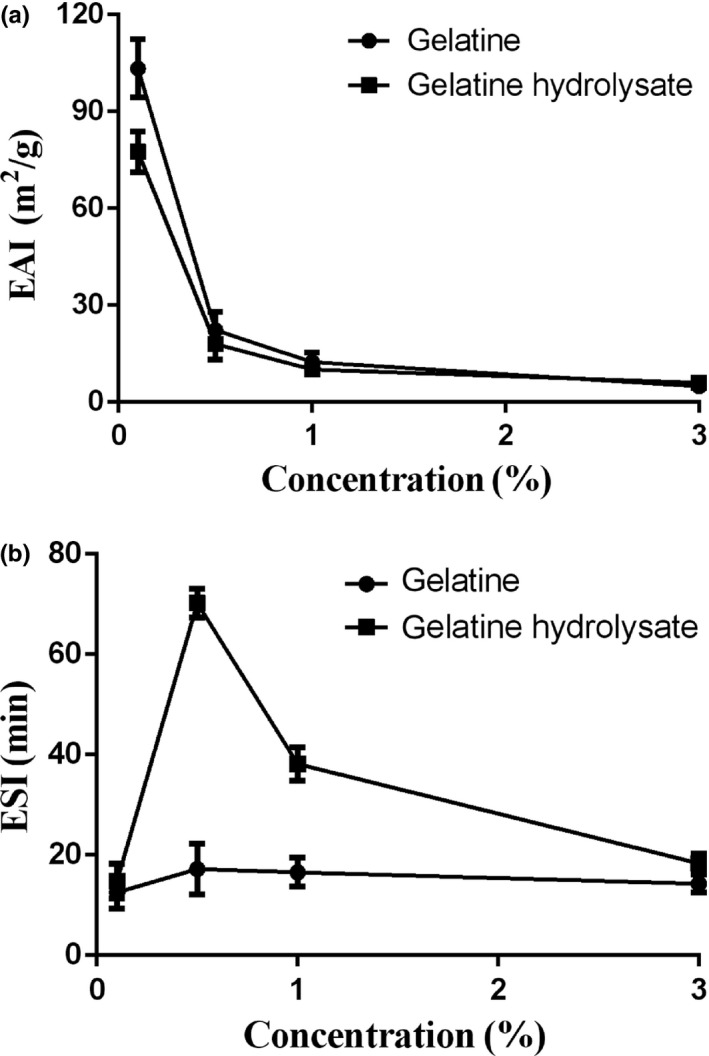
Effect of concentration on (a) emulsifying activity index (EAI) and (b) emulsion stability index (ESI) of deer antler base gelatine and its hydrolysate; bars represent standard errors (*n* = 3)

The EAI and ESI are commonly used to evaluate emulsifying properties. Figure 6 shows the effect of concentration on the emulsifying properties of the gelatine and its hydrolysate. A high EAI value corresponds to a high interfacial area in a o/w emulsion (Mazorra‐Manzano, Pacheco‐Aguilar, Ramírez‐Suárez, Garcia‐Sanchez, & Lugo‐Sánchez, 2012). The highest EAIs for both the gelatine and its hydrolysate were obtained at a 1.0 mg/ml concentration (77.57 m^2^/g and 103.36 m^2^/g, respectively). The gelatine with a DH of 3.44% had a higher EAI than the hydrolysate with a DH of 1.61%. Enzyme hydrolysis on the gelatine damaged the hydrophobic segment in the protein molecule and structurally maintained macromolecule stability (from hydrogen bonds, van der Waals forces, ionic bonds, etc.). Thus, the emulsifying ability of gelatine hydrolysate decreased, which was confirmed by the lower *S*_0_ shown in Table 1. Large peptides can expand and adapt to interfacial tension (Gbogouri, Linder, Fanni, & Parmentier, 2004). Therefore, increasing the DH reduces emulsification ability. Witono, Taruna, Windrati, Azkiyah, and Sari (2016) found a negative correlation between the DH and emulsifying properties, in agreement with the results of Lopes‐da‐Silva and Monteiro (2019). Increasing the concentration reduced the EAI, in agreement with the results of Rajabzadeh et al. (2018).

At 1.0–30.0 mg/ml concentrations, the gelatine hydrolysate had a higher ESI than gelatine. The emulsifying properties of a protein can be affected by numerous factors, such as enzyme specificity, differences in the molecular weight distribution (Nyo & Nguyen, 2019), or hydrophobic properties (Mutilangi, Panyam, & Kilara, 1996). In this study, the thin protein layer appeared to be the main factor promoting the stability of oil particles. The highest ESIs for the gelatine and its hydrolysate of 17.20 and 70.22 min, respectively, were obtained at a 5.0 mg/ml concentration. Beyond a certain protein concentration, the quantity of protein per unit area has been reported to cause interference (Intarasirisawat et al., 2012). As increasing the DH has been reported to enhance emulsion stability (Chalamaiah, Jyothirmayi, Prakash, & Dinesh Kumar, 2015), the increase in the gelatine hydrolysate ESI may have resulted from the higher DH of the gelatine hydrolysate than that of the gelatine. The results for the emulsifying properties found in this study are consistent with those for protein hydrolysates from the roe of rainbow trout reported by Rajabzadeh et al. (2018).

### 3.8 Antioxidant activities

**FIGURE 7:**
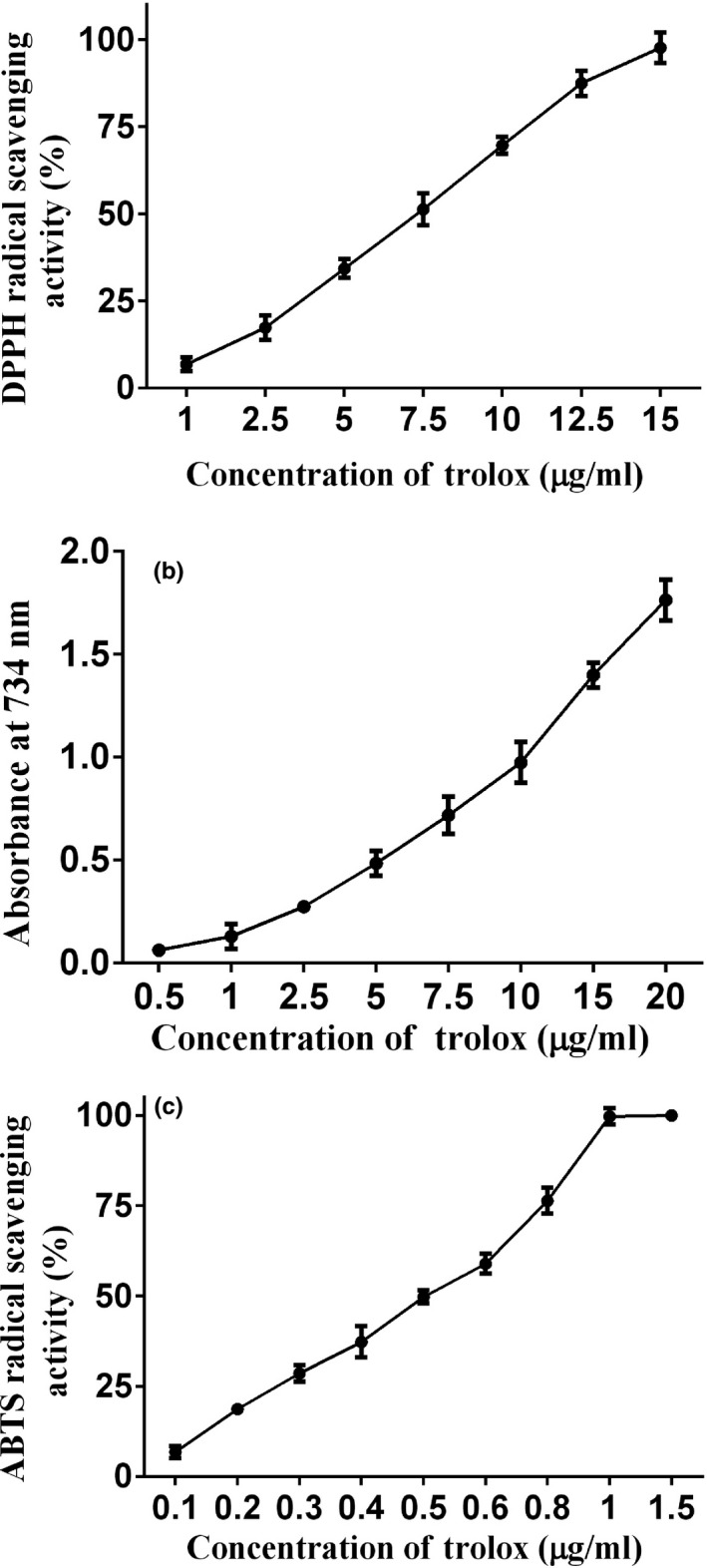
Antioxidant activity of Trolox

**FIGURE 8:**
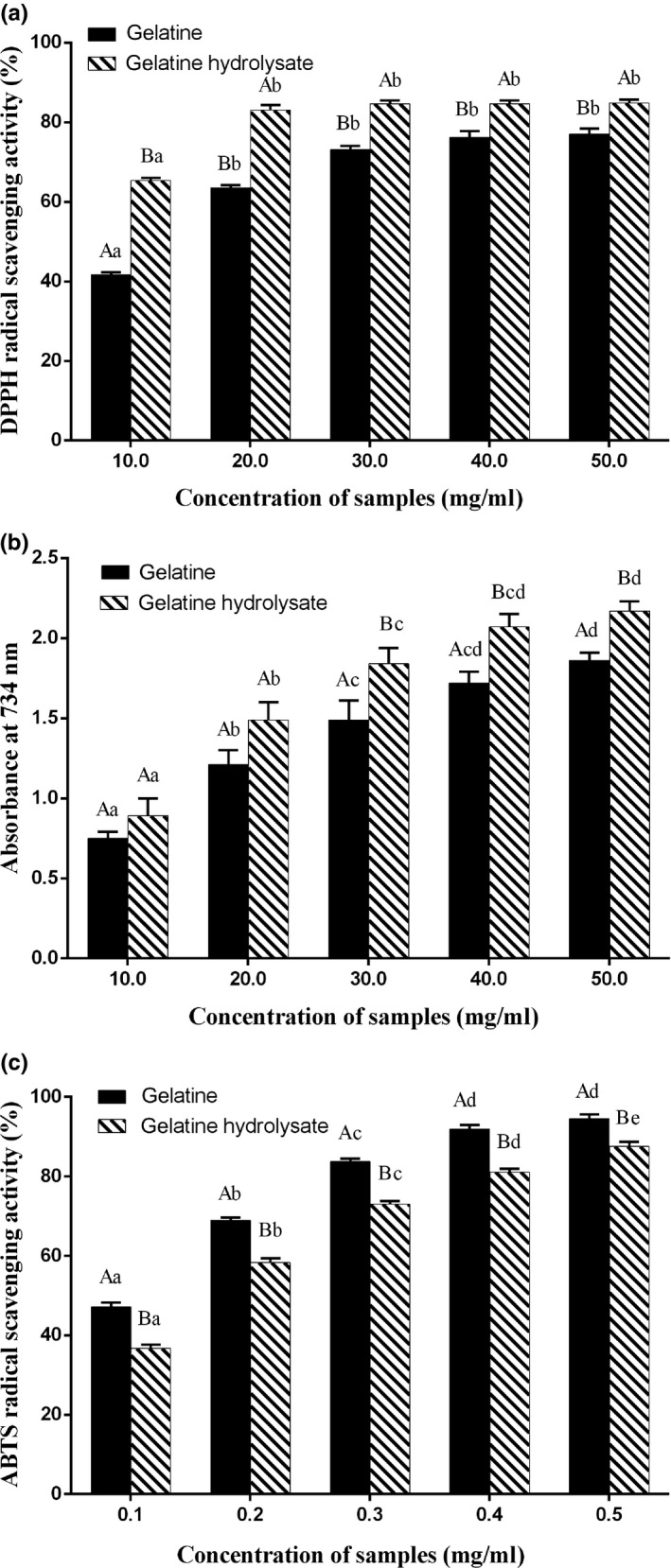
Antioxidant activity of deer antler base gelatine and its hydrolysate: (a) DPPH scavenging activity; (b) FRAP scavenging activity; and (c) ABTS scavenging activity; bars represent standard errors (*n* = 3); different lowercase (uppercase) letters within same samples (concentration) indicate significant differences (*p* < .05)

The antioxidant activity of Trolox was used as a positive control, as shown in Figure 7a–c. Trolox exhibited excellent performance for scavenging DPPH, FRAP, and ABTS activity. Both the gelatine and its hydrolysate exhibited DPPH radical scavenging activity. As shown in Figure 8a, the antioxidant activity of the gelatine and its hydrolysate increased with concentration. It is likely that the gelatine and its hydrolysate contain peptides or amino acids with electron‐donating properties that can terminate DPPH radical chain reactions (Jemil et al., 2014). Protein hydrolysates/peptides, hydrophobicity, or hydrophobic amino acids have been correlated with DPPH radical scavenging activity (Li, Jiang, Zhang, Mu, & Liu, 2008). Amino acids, such as histidine, methionine, cysteine, and phenylalanine, may have contributed to DPPH scavenging (Jemil et al., 2014). Zheng, Hao, Weng, and Ren (2019) strongly correlated (correlation coefficient ≥.98) for glycine, tyrosine, and phenylalanine with DPPH free radical scavenging activity, which is consistent with our amino acid composition results in Table 1. The gelatine hydrolysates at all concentrations exhibited higher DPPH scavenging activity than gelatine (*p* < .05). These results are consistent with those of Rajabzadeh et al. (2018), wherein a DPPH scavenging activity increased with the hydrolysate concentration. Figure 8b shows the same patterns for the FRAP assay as for the DPPH assay. Both the gelatine and its hydrolysate showed FRAP radical scavenging activity. The gelatine hydrolysates had a higher Fe^3+^ reducing capacity than gelatine (30–50 mg/ml, *p* < .05). The increase in FRAP activity has been correlated with an increased quantity of the lower molecular weight moiety from hydrolysis by trypsin (Akhilesh, Chatli, Kumar, & Mehta, 2019). Enzymatic digestion has been reported to produce small peptide molecules, which have higher reducing power than larger ones (Khantaphant, Benjakul, & Ghomi, 2011). The gelatine and its hydrolysate exhibited a high ABTS free radical scavenging activity of 0.1–0.5 mg/ml, as shown in Figure 8c. The antioxidant activity of the gelatine and its hydrolysate increased with the concentration, reaching 94.51% and 87.49%, respectively, at a 0.5 mg/ml concentration. The ABTS free radical scavenging activity depends entirely on the ability of antioxidant peptides to participate in hydrogen transfer (Olagunju, Omoba, Enujiugha, Alashi, & Aluko, 2018).

The antioxidant capacities of the gelatine and its hydrolysate were expressed in terms of the Trolox equivalent antioxidant activity (TEAC, mg Trolox/g sample, corresponding to the linear part of each curve), as shown in Table 3. The TEAC of ABTS was 3.87 ± 0.06 mg Trolox/g and 5.24 ± 0.09 mg Trolox/g for the gelatine and its hydrolysate, respectively. Thus, both samples showed excellent antioxidant activity in the ABTS assay, but weak free radical scavenging abilities in the DPPH and FRAP assays. These results show that the gelatine hydrolysate had higher antioxidant properties after trypsin modification, especially in terms of scavenging ABTS radicals.

**TABLE 3:** Antioxidant activity of deer antler gelatin and gelatin hydrolysate in vitro

	TEAC (mg Trolox/g sample)		
	DPPH	ABTS	FRAP
Gelatin	0.71 ± 0.03^a^	3.87 ± 0.06^a^	0.80 ± 0.05^a^
Gelatin hydrolysate	1.19 ± 0.01^b^	5.24 ± 0.09^b^	1.02 ± 0.08^b^

Values are means ± standard deviation. Different letters in the same row indicate significant differences (*p* < .05).

Abbreviation: TEAC, Trolox equivalent antioxidant activity.

We formulated three hypotheses to explain the results above. (a) The amino acid composition of gelatine or its hydrolysate was closely related to the antioxidant activity. (b) Trypsin hydrolysis decomposed the gelatine macromolecules into small segments that could easily access free radical sites. (c) The decomposition and aggregation of these segments in the hydrolysate resulted in the exposure of more active sites. Verma, Chatli, Kumar, and Mehta (2018) explained the low antioxidant activity of nonhydrolysed proteins in terms of molecular compactness and suggested that enzymatic digestion disrupts the natural protein structure, leading to the opening and formation of peptides or amino acid residues that react with free radicals/oxidants. However, the exact mechanism for peptide antioxidant action and whether a synergistic interaction affected antioxidant activity was not elucidated and requires further investigation. The animal origin of deer antler base gelatine and its hydrolysate results in an amino acid composition close to that of human body. Thus, the peptide fragments resulting from enzymatic hydrolysis are easily accepted by the human body. In addition, the biological activities of deer antler base result in anti‐inflammatory, antifatigue, and antistress properties (Wu et al., 2013) with potential application in functional foods and cosmetics.

## 4 CONCLUSION

Trypsin hydrolysis had the following effects on deer antler base gelatine: broadening of the polydispersity, a reduction in the zeta potential, enhanced thermal stability, decreased surface hydrophobicity, and increased emulsifying stability. These results suggest that trypsin treatment may change the functional properties of deer antler base gelatine. The obtained hydrolysate exhibited enhanced DPPH, FRAP, and ABTS free radical scavenging ability. The results showed that deer antler base gelatine hydrolysates are potential sources of bioactive peptides with good functionality with application to the food, pharmaceutical, and cosmetic industries. Further studies are required in this area.

## CONFLICT OF INTEREST

The authors declare no conflict of interest.
